# Sterylglucosides in Fungi

**DOI:** 10.3390/jof8111130

**Published:** 2022-10-26

**Authors:** Nivea Pereira de Sa, Maurizio Del Poeta

**Affiliations:** 1 Department of Microbiology and Immunology, Stony Brook University, Stony Brook, NY 11794, USA; 2 Institute of Chemical Biology and Drug Discovery (ICB&DD), Stony Brook, NY 11794, USA; 3 Division of Infectious Diseases, School of Medicine, Stony Brook University, Stony Brook, NY 11794, USA; 4 Veterans Administration Medical Center, Northport, NY 11768, USA

**Keywords:** glycolipid, sterylglucoside, sterol, sterol glucosyltransferase, ß-glucosidase, sterylglucosidase, fungi, *Cryptococcus*, *Candida*, *Aspergillus*

## Abstract

Sterylglucosides (SGs) are sterol conjugates widely distributed in nature. Although their universal presence in all living organisms suggests the importance of this kind of glycolipids, they are yet poorly understood. The glycosylation of sterols confers a more hydrophilic character, modifying biophysical properties of cell membranes and altering immunogenicity of the cells. In fungi, SGs regulate different cell pathways to help overcome oxygen and pH challenges, as well as help to accomplish cell recycling and other membrane functions. At the same time, the level of these lipids is highly controlled, especially in wild-type fungi. In addition, modulating SGs metabolism is becoming a novel tool for vaccine and antifungal development. In the present review, we bring together multiple observations to emphasize the underestimated importance of SGs for fungal cell functions.

## 1. Introduction

The biological membrane’s major lipids are glycerophospholipids, sphingolipids, and sterols. The complex and dynamic organization of these lipids determines membrane fluidity, permeability, and the optimal functioning of cells [1]. Sterols are hydrophobic isoprenoid-derived lipids present in plants, animals, fungi, protozoa, and some bacteria [2]. They are clustered with sphingolipids to form lipid rafts, where various enzymes are located to activate signaling pathways regulating numerous biological processes such as phagocytosis, stress tolerance, and biogenesis of lipid droplets, to cite a few [3,4,5,6,7]. Although lipid rafts are present in all eukaryotic plasma membranes, they are more common in fungal cells compared to mammalian cells because ergosterol (fungal sterol), is a better raft-former than cholesterol (mammalian sterol) [7,8].

**Figure 1:**
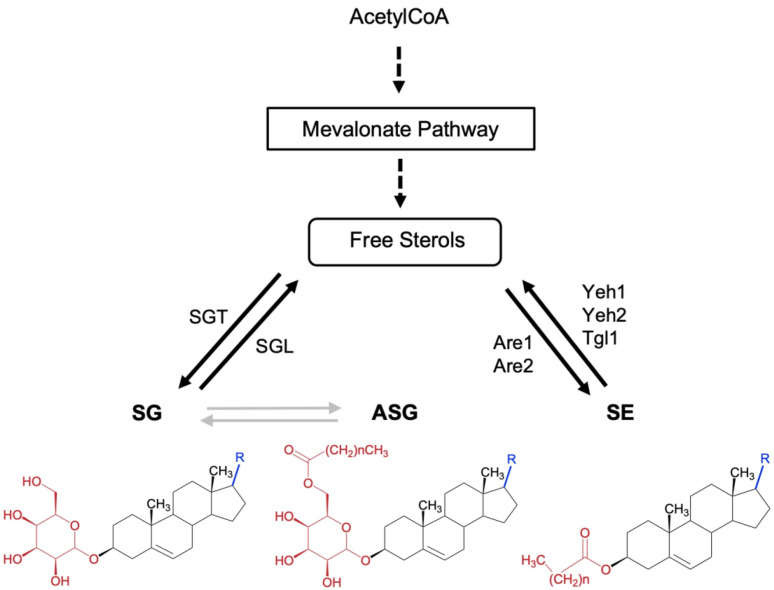
Schematic overview of conjugated sterol metabolism in fungi. The chemical structure of free and conjugated sterols sterylglucosides (SG), acyl Sterylglucosides (ASG), and sterol esters (SE). Dashed arrows indicate multiple steps. The position of the enzymes sterol glycosyltransferase (SGT) and sterylglucosidase (SGL) is indicated. SGs are characterized by having a sugar linked to the C3 hydroxyl group of the sterol moiety through a β-glycosidic bond. ASGs are derivatives of SGs in which the hydroxyl group of the C6 position of the sugar moiety is acylated by sterol glycoside acyltransferase (SGA), however, no SGA has been characterized in fungi to date. Gray arrows represent enzymatic pathways still to be characterized. In SEs the hydroxyl group at the C3 position is esterified with a fatty acid by sterol O-acyltransferases, named Are1 and Are2 in *Saccharomyces cerevisiae*, which catalyzes the formation of sterol esters and act in concert with the sterol ester hydrolases Yeh1, Yeh2, and Tgl1.

In addition to existing as free form lipids, sterols (fungal or mammalian) can also be conjugated to form sterol esters (SEs), sterylglucosides (SGs), or acyl sterylglucosides (ASGs) (Figure 1). SEs have been studied mainly in plants and yeasts, and they are produced in the first steps of lipid droplet biogenesis [9,10]. SEs can be present in the form of soluble lipoprotein complexes facilitating sterol transport within cells and between tissues; one example is cholesterol, which is transported in the blood as cholesteryl esters in the form of low-density lipoproteins (LDL) [11]. Most of the literature on SGs is related to plants; however, interest in fungi has been emerging in the past several years [12,13]. SGs are essentially hydrophilic conjugates that confer the ability to form water-soluble structures and be incorporated in cellular membranes. As such, they can modify physicochemical properties of cell membranes, such as cellular mobility, fluidity, permeability, hydration, and phase behavior, and, interestingly, alter the immunogenicity of the cells [14,15,16]. Finally, SGs can be acylated at the C6 of the sugar moiety with fatty acids forming ASGs increasing the hydrophobicity of the membrane [17]. ASGs are widely distributed in nature, however, most of the studies focus on plants, in which ASGs are frequently isolated in complex mixtures with SGs and themselves required for plant development and response to pathogens [18].

Compared to free sterols, (ergosterol or cholesterol), glycosylated sterols such as SGs exist at extremely low levels in living organisms. Thus, the studies of these lipids have been limited and difficult to perform. However, in recent years the advent of new and more sensitive mass spectrometry has allowed better analysis of these lipids in cells [19,20,21,22]. More importantly, genetic approaches (mostly in fungi) permitted the generation of mutant cells that accumulate SGs, which prompted the examination of their functions in biology and physiopathology of fungal organisms [23,24]. In this review, we summarize some of the enigmatic biological functions of SGs in fungi.

## 2. Sterylglucosides in Fungi

Ergosterol is the major sterol component of fungal membranes, and it is the main sterol specie used to make SGs. Thus, fungal cells mostly produce ergosterol 3β-D-glucoside [23].

The composition of sterols in SGs reflects the number of free sterols in each organism that can be identified by LC-MS. Since ergosterol is the major sterol component of fungal membranes, the cells mostly produce ergosterol 3β-D-glucoside [23]. Plants, however, produce a complex mixture of sterols that mainly differ in the nature of the side chain at position C17 and the number and position of double bonds in the rings or the lateral chain generating various and unique sterols and SGs. The major sterol bases of SGs in plants are sitosterol, stigmasterol and campesterol [18].

Ergosterol 3β-D-glucoside is present at a low, almost undetectable, level in fungal wild-type cells of *Cryptococcus*, *Candida*, *Saccharomyces*, *Neurospora,* and *Pichia* [25,26,27,28]. This is also the case in plant cells where SGs correspond to only ~10% of the sterol content in the cells, although this level can be quite different among plant species and tissues [29,30].

From the sterol counterpart, SGs in fungi can be formed with ergosterol and with other intermediates in the ergosterol biosynthetic pathway depending on the specie. The level of SGs increases when cells are exposed to certain stress conditions, such as cold or heat, and this happens in plant, fungal, or mammalian cells, [26,31,32]. However, the physiological relevance of this increase is still unclear. For instance, in yeasts, certain strains of *Kluyveromyces lactis* usually do not produce detectable amounts of SGs, except strain M-16, isolated from raw milk and milk products, in which the level of SGs (mostly made by ergosterol and dihydroergosterol) is surprisingly high, reaching 27% of the total sterol-derived lipids [33]. Because neither ergosterol nor dihydroergosterol are present in milk, these SGs are synthesized by *K. lactis* M-16. In plants, there are also some remarkable exceptions of surprisingly high SGs levels in plants of the genus *Solanum*, whereas in tomato fruit SGs and ASGs represent more than 85% of total sterol content [34]. SGs also represent the major sterol fraction in phloem sap collected from *Phaseolus vulgaris* and *Nicotiana tabacum*; however, the ecological implications of it to phloem sap-feeding insects are still unknown [35]. In animals, SG levels are usually low and whereas no specie appear to naturally be an exception to it, a higher expression is observed when induced by heat shock treatment as observed in human fibroblasts in vitro [31].

Regarding the sugar moiety, SGs mostly contain D-glucopyranose in a β-anomeric configuration in plant, fungal, mammalian, and in certain bacterial cells (e.g., *Borrelia*) [14,31,36,37,38,39]. In addition to β-glucosides, SGs with α-anomeric configuration have been found in *Helicobacter pylori*, which, upon uptake, converts mammalian cholesterol into cholesteryl 6′-O-acyl-α-D-glucopyranoside [40,41]. Several examples of SGs formed with sugar species other than glucopyranosides are reported in the literature, for example, galactosylated cholesterol in vertebrates’ brain, β-D-glucuronopypanoside in human liver, and α-mannopyranoside in *Candida albicans* [42,43,44,45]. The fungal SG formed with glucose will be the focus of this review in the following sections.

## 3. SG Metabolism

The formation of sterylglucosides from sterols in plants, fungi, and bacteria uses UDP-glucose as a sugar donor [14,46]. The synthesis of SG in fungi involves a sterol glucosyltransferase (SGT) whereas a sterylglucosidase (SGL) enzyme is responsible for their breakdown. Contrastingly, in mammalians no SGT and SGL have been identified to date, however, there is evidence that glucocerebrosidases (GBA) use glucosylceramide (GlcCer) as a source of glucose and catalyze the transglycosylation to cholesterol β-glucoside. In contrast to fungi where a different enzyme is responsible for breaking down SGs, in mammalian cells GBA is also able to breakdown cholesterol β-glucoside into free sterol and glucose, particularly when there is a decrease in the free cholesterol availability intracellularly [42,47]. Having one enzyme to perform two distinct (and opposite) reactions and keeping a low and constant SG content suggest that the level of intracellular free and conjugated cholesterol is tightly regulated in mammalian cells. Fungal SGT and SGL will be discussed in the sections to follow.

### 3.1. Sterol Glycosyltransferase (SGT)

Glycosyltransferases (GTs) belong to a large family of enzymes that catalyze the transfer of an activated glycosyl donor to specific acceptor molecules, forming glycosidic bonds [48]. GTs have been classified into 115 families based on sequence identity (CAZy: www.cazy.org accessed on 10 August 2022). Most of the GT family 1 members are defined by the presence of a carboxyl-terminal consensus sequence termed as the signature motif involved in the interaction of the enzyme with the activated sugar donor, which can be identified in the enzyme sequences of animals, plants, fungi, and bacteria (reviewed in [49]).

The sterol glycosyltransferases (SGTs) are among the members of GT family 1 that transfer the sugar from UDP-glucose to a sterol with the formation of a glycosidic bond between the anomeric carbon of glucose and the 3-hydroxyl group of the sterol [49]. SGTs can act on several sterols such as ergosterol, cholesterol, sitosterol, campesterol, and stigmasterol, depending on their sources [17].

The first SGT was first purified from oat, and later, using amino acid sequence similarities from Ugt80A1 and Ugt80A2, previously identified in plants, several SGT enzymes were described in fungi, such as *Saccharomyces cerevisiae*, *C. albicans*, *Pichia pastoris*, and *Dictyostelium discoideum* [28,50]. Structurally, SGTs possess a three-domain architecture comprised of a GRAM domain and a pleckstrin homology (PH) domain at the N-terminus, and a catalytic domain (Glyco_transf_28 and UDPGT) at the C-terminus. The GRAM domain is essential for proper protein association with its target membrane, the PH domain exhibits lipid-binding activity, and the catalytic domain transfers glucose into sterol(s) [51]. Chen et al. [51] elucidated the first crystal structure of a fungal SGT (UGT51), a membrane-associated protein from *S. cerevisiae*. These authors obtained the crystal structure of the UGT51 glycosyltransferase domain and the complex structure with the sugar donor UDP-glucose. According to structural predictions, the sterol moiety sits at the UDP-glucose binding site, and the pocket is mainly formed by hydrophobic residues [51].

Furthermore, SGTs are reported to play several biological functions depending on the fungal specie. For example, in the methylotrophic yeast *P. pastoris,* SGT is involved in vacuole-dependent selective degradation of peroxisomes in response to glucose or ethanol, where ergosterol 3β-D-glucoside accumulates under stress conditions such as heat shock or excess ethanol [52,53]. Differently, in the *Yarrowia lipolytica* the UGT51 enzyme is not required for pexophagy, but for utilization of decane [53]. Pexophagy will be more discussed later in this review. Moreover, a SGT gene homolog in *Colletotrichum gloeosporioides* is induced by hard surface contact of the conidia [54]. These are a few examples pointing out the diversity of functions that require SG synthesis in fungi, besides that the ubiquitous presence of SG and SGT among fungi suggests that they might be involved in many other essential functions yet to be discovered.

### 3.2. Sterylglucosidase (SGL)

While working on the characterization of the gene involved in the catabolism of the sphingolipids, we discovered CNAG_05607 as the gene homolog to EGCrP1, which is the glucosylceramidase active in neutral and alkaline pH [55]. We initially thought CNAG_05607 was a second glucosylceramidase, but our biochemical analysis suggested that CNAG_05607 was a sterylglucosidase. In fact, CNAG_05607 from either *Cryptococcus neoformans* [23], and its homologs from *Aspergillus fumigatus* [24], metabolize SGs and not GlcCer, as claimed improperly by an early paper which uses a different, short chain, non-physiological GlcCer as a substrate [25]. Our biochemical studies were confirmed genetically. In fact, deletion of Sgl1 in *Cn* does not cause any change in the level of endogenous GlcCer but rather a dramatic accumulation of SGs, as measured by thin layer chromatography (TLC) [23] and confirmed by either gas chromatography-mass spectrometry [23] and by liquid chromatography-mass spectrometry [23]. Thus, we named CNAG_05607 sterylglucosidase 1 (Sgl1), as the first sterylglucosidase ever isolated from any living organism [23]. Both *C. neoformans* EGCrP1 and Sgl1 are hydrolases that belong to GH family 5, which is one of the largest of all CAZy GH families and includes endoglucanase, endomannanase, β-glucosidase, and β-mannosidase.

Sgl1 is a cytosolic β-glucosidase that is universally conserved among fungi (Table 1) and does not have a homolog in mammals. We have recently elucidated the first SGL structure, the *C. neoformans* Sgl1 [56]. Sgl1 structure revealed two domains comprising a catalytic domain with a central TIM barrel and a C-terminal β-sandwich domain. The general architecture of this enzyme conserves some similarity to the bacterial endoglucoceramidase II (EGCase II) from *Rhodococcus* sp. and the human glucosylceramidase, however, the larger catalytic domain of Sgl1 has additional structural elements forming a cap-like region above the TIM barrel that creates an enclosed Y-shaped cavity [56,57,58].

The *C. neoformans* Sgl1 crystal structures show that the active site pocket of the enzyme has a Y-shaped cavity, limiting binding to a single glucose moiety and it is unable to accommodate the natural/physiological fungal GlcCer, because the long hydrophobic tail of fungal GlcCer (C18-C9methyl GlcCer) does not fit in the active site, whereas the short chain non-physiological GlcCer (C6-GlcCer), used in the studies by Watanabe et al. [25], does fit the active site of Sgl1 [56]. Thus, our biochemical, genetic, and now structural data conclusively demonstrate that Sgl1 is a sterylglucosidase only, explain the controversial data published by other authors [25], and emphasize that biochemical enzymatic characteristics about substrate specificity should be attributed only upon testing natural/physiological and not artificial/non-physiological substrates.

There is no structure available for the *C. neoformans* EGCrP1, which is a glucosylceramidase. Interestingly, the analysis of the structural model (H1AE12) available on the Alphafold database suggests that the active site of EGCrP1 has a very similar architecture of Sgl1, except for its transmembrane domain, which is not present in Sgl1. Whereas it is clear why Sgl1 cannot use GlcCer it is unclear why EGCrP1 does not use SGs as a substrate, based on modeling studies.

Other SGLs from fungi have been expressed and biochemically tested but still without structural elucidation. Watanabe et al. [27] described EGH1, a Sgl1 homolog, in *Saccharomyces cerevisiae*. These authors assessed that the purified recombinant Egh1 hydrolyzed various β-glucoside substrates including ergosterol 3β-D-glucoside, cholesterol 3β-D-glucoside, sitosterol 3β-D-glucoside, and other artificial substrates: para-nitrophenyl β-glucoside, 4-methylumberifellyl β-glucoside, and C6-NBD-glucosylceramide. Similarly, to Sgl1, the disruption of EGH1 in *S. cerevisiae* BY4741 (Δ *egh1*) resulted in the accumulation of ergosterol 3β-D-glucoside, and fragmentation of vacuoles [27].

## 4. SG Modulation as a Tool for Vaccine and Drug Development

### 4.1. Cryptococcus

Cryptococcosis is a life-threatening fungal disease caused by *Cryptococcus neoformans*, an environmental fungal pathogen that infects humans via the respiratory tract. It is caused mainly by *C. neoformans* and *C. gattii*. *C. neoformans* is the most prevalent species and is predominantly associated with HIV or other immunocompromising conditions, whereas *C. gattii* infections have been also described in immunocompetent individuals [59,60].

Due to *C. neoformans* worldwide distribution, it is proposed that humans are exposed to this fungus since childhood [61,62] and that, upon primary infection, the host contains the yeast cells inside a lung granuloma [63,64,65,66,67,68,69,70,71,72,73,74,75,76]. There is evidence supporting this idea showing that fungal strains from patients developing cryptococcal meningoencephalitis are identical to those strains isolated earlier from the same asymptomatic patients [77,78,79]. In contrast, other investigators refute this possibility claiming that humans are constantly exposed to environmental strains and, when an individual is affected by the same isolate found in their body years before, it simply means that he/she inhaled the very same strain [80,81]. This is possible but does not consider the enormous genetic variability of cryptococcal strains present in the environment [82,83,84]. Thus, the chances to inhale genetically identical strains years apart are really small. It is our opinion that primary infection, granuloma formation, and eventual reactivation upon immunosuppression likely reflect the stages of this disease. Cryptococcal meningitis is the second leading cause of mortality in AIDS patients, only behind tuberculosis [85].

The current antifungal arsenal is associated with adverse effects and resistance, evidencing the urgency for the development of both therapeutic and prophylactic tools. Until the present moment, no fungal vaccine against cryptococcosis has been approved by the FDA for clinical trials, although several preclinical studies had assessed multiple antigens and adjuvants, as well as mutant fungal cells to control cryptococcosis and other fungal infections (reviewed in [86,87]). However, most of them have not been performed in animal models of immunodeficiency, which is the most frequent state of individuals that will be affected by serious fungal diseases.

The vaccine development with mutants lacking SGL enzyme has been the focus of our research group in the past years. The genetic ablation of Sgl1 resulted in an accumulation of ergosterol 3β-D-glucoside in *C. neoformans* or *Aspergillus fumigatus* [23,24,25,27]. The immunological function of SGs was first suggested in 1996 by Bouic and colleagues [88]. It was reported that the immune response and, in particular, the response of murine T-helper cells in vitro was affected by the administration of certain plant sitosterolin (a mixture of sitosterol/sitosterol β-glucoside). More importantly, the secretion of Th1 cytokines, such as IL-2 and IFN-γ, was increased [88]. Later, Lee et al. [89] found that mice infected with *Candida albicans* and treated with plant sitosterol β-glucoside survived longer than untreated mice, and splenic lymphocytes from these mice were activated compared to untreated mice. The effect of β-sitosterol glucoside on the immune response was also observed in a human study [90]. The daily administration of plant β-sitosterol glucoside (when combined with regular treatment) increased Th1 lymphocyte proliferation and promoted recovery of patients with pulmonary tuberculosis and patients suffering from allergic diseases such as rhinitis and sinusitis [89,91]. These studies suggest that sitosterol β-glucoside is a potential immune stimulator because it can shift the Th1/Th2 balance towards a more potent Th1 immune response.

Studies from our group had shown that the absence of Sgl1 renders the *C. neoformans* non-pathogenic and the vaccination with a Δ*sgl1* strain prevents secondary infections in murine models of immunocompetent or CD4^+^ T cell-depleted mice, suggesting that the accumulation of ergosterol 3β-D-glucoside is involved in the development of protective immunity [23].

Thereafter, Colombo et al. [92] demonstrated that the cryptococcal capsule is required for protection because an acapsular mutant does not protect against a secondary infection even if it accumulates ergosterol 3β-D-glucoside. In fact, although the genetic ablation of Sgl1 in the acapsular mutant Δ*cap59* (Δ*cap59*/Δ*sgl1* double mutant strain) causes an accumulation of SGs similar to the one observed in the Δ*sgl1* single mutant, it does not induce protection in a vaccination model, suggesting that ergosterol 3β-D-glucoside-related protection requires GlucuronoXyloMannan (GXM), the main components of the capsule. However, which specific capsule components (glucuronic acid, xylose, or mannose) are required for protection is yet to be solved.

On the immune mechanism of protection, Normile et al., [93] found high levels of ergosterol 3β-D-glucoside in the lungs post-vaccination with *C. neoformans* Δ*sgl1* in immunocompromised mice coinciding with a robust pro-inflammatory environment with increased leukocyte recruitment to the lungs. Interestingly, these authors observed that even under immunosuppression the mice eliminated the mutant cells from the lungs when monocytes, macrophages, and/or neutrophils, as well as B or CD8^+^ T cells, were depleted, and the animals were still fully protected against a subsequent wild-type challenge [93]. They found that lung tissue γ/*δ* T cells are stimulated *C. neoformans* Δ*sgl1*, even in absence of CD4^+^ T cells, and they are responsible for inducing protection against a secondary infection in mice.

The initial characterization of *C. neoformans* Δ*sgl1* revealed that the mutant does not have an altered growth at alkaline or acidic pH or a defect in melanin production or a different capsular size [23]. Also, there were no differences among wild-type (WT), Δ*sgl1* or Δ*sgl1* + *SGL1* reconstituted strain when cells were grown intracellularly (within macrophages) or when they were exposed to hydrogen peroxide or nitrosative stress [23]. We also found no difference in urease activity or in the secreted phospholipase B1 activity. However, when the Δ*sgl1* mutant is incubated in physiological media, such as Yeast Nitrogen Base (YNB) and low oxygen (~5–10%) it cannot grow, and, eventually, it dies [56]. These results suggest that accumulation of SGs is not tolerated by *C. neoformans* cells when exposed to in vitro conditions mimicking physiological host environments.

Thus, we reasoned to obtain the same benefits by targeting Sgl1 pharmacologically. In fact, we found that by inhibiting Sgl1 with a specific inhibitor we can reproduce the same phenotype of ergosterol 3β-D-glucoside accumulation and reduces the virulence of *C. neoformans* wild-type cells, preventing brain dissemination in the murine model [56].

**Figure 2:**
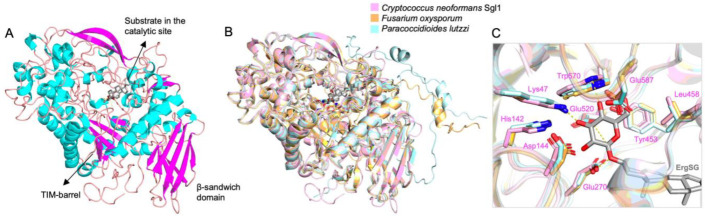
SGL1 homologs structural comparison. (**A**) *Cryptococcus neoformans* Sgl1 structure (PDB: 7LPO) with ergosterol 3β-D-glucoside (gray) docked in the active site. (**B**) *Fusarium oxysporum* (orange) and *Paracoccidioides lutzii* (cyan) Alphafold models superposed on Sgl1 (pink) structure. (**C**) The residues on the glucose binding site are conserved in a similar position in *F. oxysporum* and *P. lutzzi* models. Sgl1 residues are shown in pink.

Since Sgl1 homologs are found in other fungi, including yeast, mold, and dimorphic fungi (Table 1), one could presume that a similar phenotype may occur widely among fungi and Sgl1 could be broadly targeted by a specific inhibitor. This idea is reinforced when analyzing Sgl1 homologs predicted structure models of two representants of mold and dimorphic pathogenic fungi, *Fusarium oxysporum,* and *Paracoccidioides lutzii,* respectively, in comparison with *C. neoformans* Sgl1 (Figure 2). These models with more than 90% confidence in the active site show a very similar structural architecture with two domains and well-conserved active site residues, suggesting that it would be possible to develop a broad-spectrum anti-SGL1 inhibitor.

### 4.2. Aspergillus

The fungus from the *Aspergillus* genus is ubiquitous in the environment and can cause a wide spectrum of illnesses from non-invasive allergic forms to deadly invasive aspergillosis (IA) depending on the host immune state [94,95]. After the inhalation or inoculation with *Aspergillus* conidia, an infection may develop locally or disseminate to adjacent or distant sites, particularly in those receiving immunosuppressive therapy or who are neutropenic following bone marrow transplantation or under chemotherapy [96,97,98]. Under this immune status, IA outcome is related to high mortality rates despite the availability of antifungal therapy [99].

The metabolism of SG in several molds and the identification of SGT homologs have been described [100,101,102]. Recently, the Sgl1 from *Aspergillus fumigatus* (SglA) was identified and the mutant lacking SglA exhibited the same phenotype of SG accumulation as previously observed in *C. neoformans* and *S. cerevisiae* [24,25,27].

Fernandes et al. [24] demonstrated that similarly to *C. neoformans* the *A. fumigatus* Δ*sglA* strain has impaired virulence and is non-pathogenic in primary infection in mice. Animals vaccinated with live or heat-killed *A. fumigatus* Δ*sglA* conidia exhibited complete protection against a subsequent *A. fumigatus* wild-type challenge.

These results in *Aspergillus* validated previous studies in *Cryptococcus* and suggest that SGs most likely act as “adjuvants” for fungal antigens. Thus, we encourage a wide exploration of SGs metabolism and the effect of its accumulation on other pathogenic fungi. Furthermore, *A. fumigatus* Δ*sglA* exhibited increased levels of ergosterol 3β-D-glucoside after 12 h of growth coinciding with the peak in ergosterol 3β-D-glucoside accumulation and a significant delay in hyphal growth, which is hypothesized that the excessive amount of ergosterol 3β-D-glucoside in the membrane might impair the establishment of the cell polarity axis and delay tissue invasion in the host [24]. It will be simply exciting to find a specific inhibitor of SglA.

### 4.3. Candida

*Candida* species are dimorphic opportunistic human pathogens that exist as a harmless commensal in healthy individuals in the skin, gastrointestinal and genitourinary tract [103,104,105]. However, when the microbiota is altered, or the individual becomes immunocompromised it can cause various diseases, ranging from superficial infections to severe disseminated infections [106,107,108]. Several distinct *Candida* species cause human disease, but mostly invasive candidiasis is caused by *Candida albicans*, which is the major specie responsible for high mortality rates in humans; other species frequently associated with human diseases are *Candida glabrata*, *Candida tropicalis*, *Candida parapsilosis*, *Candida krusei*, and *Candida auris* [109,110,111,112,113].

*Candida albicans* is capable of forming highly drug-resistant biofilms in the human host (reviewed in [114]). In the biofilm structure, the ability of *Candida* cells to alter lipid composition is a crucial adaptation for biofilm development and also influences the antifungal resistance [115]. How *C. albicans* modulates its lipid profile is a question that yet is not completely understood, however more details about *Candida* plasticity are coming to light in the past years. Generally, genome plasticity happens to be the most rapid means of evolution, adaptation, and survival in most microbes [116]. *Candida* species exhibit extensive phenotypic plasticity; they can grow as single-cells or multicellular and can undergo epigenetic switching between alternative cell states [116,117]. The yeast-hyphal transition is essential for adhesion, tissue invasion, biofilm formation, phagocyte escape, and pathogenesis [106,118].

In addition, the epigenetic switching between white and opaque causes them to require different stimuli to undergo filamentation [119]. Generally, the default white state is assumed during diseases since the cell filamentation is enhanced at the human body temperature, while opaque cell filamentation is inhibited at this temperature and is optimal at 25 °C [117]. In particular, *Candida* filamentation in the white state occurs in response to diverse stimuli, such as high temperature, neutral pH, and nutrient starvation, whereas none of these seem to induce filamentation in opaque state [117,118].

Interestingly, there are differences in the lipid composition between white and opaque cells involved in the contents of free sterols and derivatives of sterols. Ghannoum and Swairjo [120] observed that white cells contained higher proportions of free sterols than opaque cells, while opaque cells contained nearly 2.5 times higher amounts of SGs and SEs. Interestingly, opaque cells have higher SG levels and the association with less virulence, similar to *C. neoformans* and *A. fumigatus*, suggesting that once more the modulation of SG levels has a direct influence on fungal virulence, thus exploring SG-related tools for candidiasis prophylaxis and therapy might be possible.

Although SGL homologs are present in *Candida* species (orf19.4031), to this moment the sterylglucosidase in *Candida* has not been characterized. Apparently, the orf19.4031 encoding for the putative homologous of Sgl1 does not breakdown SGs [121]. In fact, when Chang et al. [121] mutated the open reading frame (orf) they did not observe accumulation of SG [121], suggesting that *Candida albicans* may have additional SGL hydrolase(s) or that the orf19.4031 does not reach the place where SGs are mostly localized. Furthermore, these authors studying solasodine-3-*O*-β-D-glucopyranoside expected that a sterylglucosidase would hydrolyze it to solasodine and glucose. Unexpectedly, testing *Candida albicans* cell lysate fractions, evidence points to the presence of a membrane β-glucosidase, and not the cytosolic Sgl1 homolog, hydrolyzing this substrate. Unfortunately, there is no further progress in this matter in identifying this membrane protein carrying this Sgl1 activity, and, thus, how SGs are hydrolyzed in *C. albicans* is unclear.

## 5. Biological Functions of SG in Fungi

### 5.1. Oxygen and pH Homeostasis

It is inevitable to make assumptions about the balance of free sterol and its glycosylated form in different cell functions. Cells have a complex network of signal cascades enabling them to metabolically adapt in response to environmental changes [122]. For instance, sterol synthesis is intrinsically related to oxygen availability since the synthesis of sterols by eukaryotes is an O_2_-intensive process; thus, its levels act as indicators of the oxygen environment of cells and also as a primary mechanism of defense against reactive oxygen species formation [123,124].

So far, no study directly investigates whether SG would participate in oxygen-related mechanisms. However, in our recent work, it was observed that the *C. neoformans* mutant Δ*sgl1* cannot grow under low oxygen and acidic pH condition while *C. neoformans* wild-type cells can survive and grow under the same conditions. Moreover, this phenotype can also be mimicked by pharmacological inhibition of Sgl1. It is possible that under this type of stress, the SG catabolism is a rapid way of obtaining free sterol and keeping the essential cell functions for longer, but in the mutant Δ*sgl1*, this contingency mechanism is not available, resulting in cell death.

Curiously, a Sgl1 homolog in *Paracoccidioides lutzii* is recognized as a negative phosphate-responsive signaling pathway (PHO), whose main role is to orchestrate the induction of PHO genes in response to phosphate starvation, but also cellular transport and carbohydrate and lipid metabolism. Additionally, the PHO pathway is tightly influenced by pH variation. Hence, PHO genes expression is essential for survival under alkaline pH. Interestingly, the sterol homeostasis pathway (SREBP) is also necessary for growth in an alkaline environment, and an elevated pH is sufficient to induce *SRE1* cleavage and activation in *C. neoformans* [125]. *SRE1*, a homolog of the mammalian sterol regulatory element-binding protein (SREBP), is activated under low oxygen, to stimulate genes required for ergosterol biosynthesis and iron uptake [126]. Perhaps, another (and faster) way to obtain ergosterol under low oxygen is to breakdown SGs. Further studies are clearly needed to understand the relationship between low oxygen adaptation and SGs metabolism.

### 5.2. Pexophagy

Autophagy is a process of bulk degradation for recycling resources under starvation conditions and a selective autophagic process is identified in organisms including mammals, plants, and fungi [127,128]. During starvation, cytoplasmic components are randomly sequestered into autophagosomes and delivered into the lysosome/vacuole to be destroyed [129]. In fungi, autophagy plays a role in hyphal growth, conidiation, oxidative stress resistance, and virulence [130]. In certain molds, autophagy is required to recycle internal components to support optimal conidiation in *A. fumigatus*, *Beauveria bassiana*, and *Magnaporthe oryzae* [131,132,133].

Pexophagy is the selective autophagic degradation of peroxisomes. These processes require SGT proteins for the autophagosome formation [134]. The need for the synthesis of SG for the membrane elongation reaction during phagosome formation is not fully understood. A hypothesis that Yamashita et al. [134] proposes is that synthesis of SG results in an asymmetric distribution of the components within the bilayer of the isolated membrane. In fact, SGs do not flip-flop as sterols do. In addition, the glucose residue protruding into the soluble phase may become a scaffold for further elongation reactions or a signal to recruit other factors, further contributing to membrane asymmetry.

*P. pastoris* SGT (PpAtg26) associated with the pexophagy process has been widely referred in the literature, especially in the degradation of methanol-induced peroxisomes [135]. PpAtg26 is recruited to the precursor of the pexophagy structure [134]. More recently, Kikuma et al. [102] demonstrated that SGT from *Aspergillus oryzae* (AoAtg26) is also required for autophagic degradation of peroxisomes mitochondria and nuclei. Moreover, deletion of this protein severely reduced conidiation and aerial hyphae formation in *A. oryzae,* similar to other molds. In *Alternaria alternata* SGT mutation led to autophagy impairment, accumulating peroxisomes, increased ROS sensitivity, and reduced virulence [136,137]. Similarly, *C. neoformans* also has impaired virulence in *Galleria mellonella* and murine models when the autophagy mechanism is disturbed [138]. In contrast, *C. albicans* autophagy defective mutant *C. albicans atg9*Δ does not require autophagy to retain its virulence in disseminated candidiasis in mice [139]. Although few, they are evidence pointing to the importance of SGT proteins for controlling the autophagic process in fungi.

## 6. Future Prospects of SG Research

There is still a long way to go in understanding the diverse roles of SG in fungi. How do SGs regulate fungal virulence? How do these lipids stimulate host immunity? How does *C. albicans* metabolize SGs? Would targeting Sgl1 improve the primary infection and possibly preventing against a secondary infection or reactivation? Those are only a few questions about these glycolipids we would like to address in the near future. In the present review, we bring together multiple observations to emphasize the underestimated importance of SG for fungal cell functions. They regulate different cell pathways to help overcome oxygen and pH challenges, cell recycling, and other membrane functions. At the same time, the level of these lipids seems to be highly controlled, especially in wild-type fungi. This makes their studies challenging but, at the same time, highly exciting.

## Figures and Tables

**Table 1:** Sterylglucosidase 1 (Sgl1) homologs in fungi. Score values obtained by “Blastp” of the *Cryptococcus neoformans* Sgl1 (CNAG_05607) amino acid sequence into the FungiDB protein database.

Fungus	Gene ID	Score
**Yeasts**		
*Cryptococcus neoformans*	CNAG_05607	1768
*Cryptococcus gattii*	I306_06474	1619
*Candida albicans*	C5_05360C_A	431
*Candida glabrata*	CAGL0L09493g	450
*Candida parapsilosis*	CPAR2_100160	424
*Candida tropicalis*	CTRG_06142	422
*Candida auris*	B9J08_001529	399
**Filamentous**		
*Aspergillus fumigatus*	Afu3g08820	546
*Fusarium oxysporum*	FOZG_03609	568
*Scedosporium apiospermum*	SAPIO_CDS4400	542
*Neurospora crassa*	NCU02233	560
*Mucor circinelloides*	HMPREF1544_10329	470
*Rhizopus delamar*	RO3G_09843	462
**Dimorphic**		
*Paracoccidioides brasiliensis*	PABG_01604	548
*Paracoccidioides lutzzi*	PAAG_08826	543
*Blastomyces dermatitidis*	BDFG_00125	563
*Histoplasma capsulatum*	HCBG_00240	570
*Coccidioides immitis*	CIMG_07690	582
*Sporothrix brasiliensis*	SPBR_06602	538

## Abbreviations

SE	sterol esters
SG	sterylglucoside
ASG	acyl sterylglucoside
GlcCer	glucosylceramide
SGT	sterol glucosyltransferase
SGL	sterylglucosidase
GT	glycosyltransferase
GBA	glucocerebrosidase
GXM	glucuronoxylomannan
IA	invasive aspergillosis
